# Safety, Tolerability, and Parasite Clearance Kinetics in Controlled Human Malaria Infection after Direct Venous Inoculation of *Plasmodium falciparum* Sporozoites: A Model for Evaluating New Blood-Stage Antimalarial Drugs

**DOI:** 10.4269/ajtmh.21-1297

**Published:** 2022-08-29

**Authors:** M. Farouk Chughlay, Stephan Chalon, Myriam El Gaaloul, Nathalie Gobeau, Jörg J. Möhrle, Pieter-Jan Berghmans, Katrin Van Leuven, Michael W. Marx, Anna Rosanas-Urgell, Julia Flynn, Emilie Escoffier, Daniel Izquierdo-Juncàs, Bastiaan Jansen, Venelin Mitov, Anne Kümmel, Jean-Pierre Van Geertruyden, Karen I. Barnes

**Affiliations:** ^1^ Medicines for Malaria Venture, Geneva, Switzerland;; ^2^ SGS Life Sciences, Antwerp, Belgium;; ^3^ ICON Clinical Research GmbH, Langen, Germany;; ^4^ Department of Biomedical Sciences, Institute of Tropical Medicine, Antwerp, Belgium;; ^5^ IntiQuan GmbH, Basel, Switzerland;; ^6^ Global Health Institute, University of Antwerp, Antwerp, Belgium;; ^7^ Division of Clinical Pharmacology, Department of Medicine, University of Cape Town, Cape Town, South Africa;; ^8^ University of Cape Town Medical Research Council Collaborating Centre for Optimizing Antimalarial Therapy, University of Cape Town, Cape Town, South Africa

## Abstract

*Plasmodium falciparum* sporozoite (PfSPZ) direct venous inoculation (DVI) using cryopreserved, infectious PfSPZ (PfSPZ Challenge [Sanaria, Rockville, Maryland]) is an established controlled human malaria infection model. However, to evaluate new chemical entities with potential blood-stage activity, more detailed data are needed on safety, tolerability, and parasite clearance kinetics for DVI of PfSPZ Challenge with established schizonticidal antimalarial drugs. This open-label, phase Ib study enrolled 16 malaria-naïve healthy adults in two cohorts (eight per cohort). Following DVI of 3,200 PfSPZ (NF54 strain), parasitemia was assessed by quantitative polymerase chain reaction (qPCR) from day 7. The approved antimalarial artemether-lumefantrine was administered at a qPCR-defined target parasitemia of ≥ 5,000 parasites/mL of blood. The intervention was generally well tolerated, with two grade 3 adverse events of neutropenia, and no serious adverse events. All 16 participants developed parasitemia after a mean of 9.7 days (95% CI 9.1–10.4) and a mean parasitemia level of 511 parasites/mL (95% CI 369–709). The median time to reach ≥ 5,000 parasites/mL was 11.5 days (95% CI 10.4–12.4; Kaplan–Meier), at that point the geometric mean (GM) parasitemia was 15,530 parasites/mL (95% CI 10,268–23,488). Artemether-lumefantrine was initiated at a GM of 12.1 days (95% CI 11.5–12.7), and a GM parasitemia of 6,101 parasites/mL (1,587–23,450). Mean parasite clearance time was 1.3 days (95% CI 0.9–2.1) and the mean log_10_ parasite reduction ratio over 48 hours was 3.6 (95% CI 3.4–3.7). This study supports the safety, tolerability, and feasibility of PfSPZ Challenge by DVI for evaluating the blood-stage activity of candidate antimalarial drugs.

## INTRODUCTION

Malaria is a life-threatening infectious disease caused by protozoan parasites, mainly *Plasmodium falciparum* and *P. vivax*. The WHO reported 241 million malaria cases in 2021 and 627,000 deaths.^1^ Effective disease control programs using artemisinin-containing combination therapies (ACTs) have contributed to a global reduction in mortality from *P. falciparum* malaria. However, there is an evolving threat of drug resistance against artemisinin derivatives, and an urgent need for the discovery and development of new antimalarial therapies.^2^

In controlled human malaria infection (CHMI), healthy human volunteers are infected with *P. falciparum* malaria parasites.^3^^,^^4^ Such studies are critical in accelerating antimalarial drug and malaria vaccine development, allowing the rapid assessment of efficacy and safety.^3^^–^^25^ They also provide the necessary data for pharmacokinetic/pharmacodynamic modeling to support dose selection for further clinical development.^9^^,^^10^^,^^16^ CHMI studies have also been used to study antimalarial immunity and other aspects of host–parasite biology.^25^^–^^31^

There are three main methods of establishing malaria infection in CHMI: intravenous administration of parasitized erythrocytes (pRBCs),^8^^–^^11^^,^^31^^–^^34^ transmission of *P. falciparum* sporozoites (PfSPZ) via bites from infected mosquitoes,^12^^–^^16^^,^^30^^,^^34^^–^^41^ or the use of cryopreserved infectious PfSPZ (PfSPZ Challenge), which are introduced via intradermal injection,^17^^,^^42^^–^^45^ intramuscular injection,^45^^–^^47^ intravenous injection,^48^ or by direct venous inoculation (DVI).^16^^–^^29^^,^^47^^–^^51^

PfSPZ Challenge using the NF54 strain by DVI has several advantages. The preparation is standardized, containing approximately 3,200 aseptic, purified, cryopreserved NF54 PfSPZ, and is manufactured according to health regulatory standards.^48^ The number of infecting parasites is controlled and consistent across experiments, producing predictable infections, reducing variability, and hence minimizing the number of volunteers required.^48^ The NF54 strain is susceptible to all standard antimalarial drugs, allowing the administration of effective rescue therapy.^52^ Unlike CHMI with PfSPZ by mosquito bite, there is no requirement for an insectary with PfSPZ Challenge by DVI. Also, there is no exposure to human blood products, as is the case with the intravenous administration of pRBCs. Thus, CHMI using PfSPZ Challenge by DVI supports efforts to expand and industrialize early phase antimalarial drug and malaria vaccine development to multiple sites, including centers in Africa.^4^^,^^18^^,^^21^^–^^23^^,^^25^^–^^29^^,^^51^

PfSPZ Challenge by DVI has been used for the evaluation of malaria vaccines,^17^^,^^18^^,^^21^^,^^25^^,^^51^ including vaccines with blood-stage activity,^18^ and to assess the chemoprophylactic activity of antimalarial drugs.^16^^,^^19^^,^^20^^,^^50^ In previous PfSPZ Challenge by DVI studies, the efficacious approved antimalarial artemether-lumefantrine has been used as rescue therapy to clear residual blood-stage parasitemia.^18^^,^^22^^,^^48^ The safety and tolerability of PfSPZ Challenge by DVI has been well established.^4^^,^^20^^,^^47^^,^^48^ However, the parasite growth kinetics of blood-stage parasitemia in malaria-naïve volunteers and parasite clearance kinetics following effective schizonticidal antimalarial drugs have not been sufficiently documented to allow the assessment of new chemical entities for their blood-stage activity.

The current study examined whether PfSPZ Challenge by DVI can be used to safely generate blood-stage parasitemia at levels and timescales comparable to those previously documented for the evaluation of blood-stage antimalarial activity in CHMI models that have established infection using intravenous administration of pRBCs or with PfSPZ by mosquito bite.^9^^–^^11^^,^^32^^,^^33^^,^^36^ To obtain the necessary data to allow the characterization of the antimalarial blood-stage activity of new chemical entities in the DVI of PfSPZ CHMI model, we evaluated parasite growth following PfSPZ Challenge by DVI and characterized parasite clearance dynamics following treatment with the approved efficacious antimalarial artemether-lumefantrine.

## MATERIALS AND METHODS

### Design and ethics.

This single-center, open-label, Phase Ib study was conducted at the SGS Phase 1 Clinical Pharmacology Unit, Ziekenhuis Netwerk Antwerpen (ZNA), Antwerp, Belgium, between February 19, 2020 and December 17, 2020. The study adhered to the Declaration of Helsinki, Guidance on Good Clinical Practice, and applicable local requirements. Ethical approval was obtained from the Commissie voor Medische Ethiek ZNA Institutional Review Board, Antwerp, Belgium. Reciprocal ethical approval was granted by the University of Cape Town, Faculty of Health Sciences, Human Research Ethics Committee, Cape Town, South Africa. All participants provided written informed consent before study participation. The study was overseen by a safety review team comprising the sponsor medical director, site principal investigator, medical monitor, malaria expert, and expert drug development physician (chairperson), who reviewed safety/tolerability and parasitemia data at prespecified time points.

**Figure 1.**
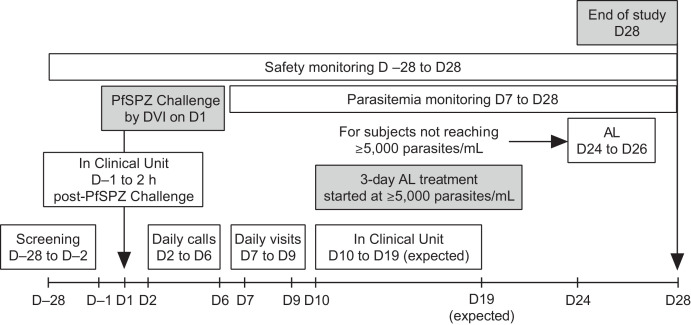
A schematic overview of the study design. AL = artemether-lumefantrine; DVI = direct venous inoculation; PfSPZ = *Plasmodium falciparum* sporozoite.

Given the exploratory nature of the study, no formal sample size calculation was performed and the sample size of 16 healthy volunteers was based on a review of published studies using intravenous administration of pRBCs.^10^^,^^11^^,^^32^ The initial plan was to enroll two cohorts sequentially of eight participants each, with cohort-specific qPCR-defined target parasitemia levels, that is, ≥ 5,000 parasites/mL in cohort 1 and ≥ 10,000 parasites/mL in cohort 2. However, the study protocol allowed for modification of the cohort 2 parasitemia targets. After a review of data from cohort 1 by the safety review team, it was decided to maintain the target parasitemia of ≥ 5,000 parasites/mL in cohort 2. A schematic overview of the study design is shown in Figure 1.

### Participants.

Eligible participants were males or females, aged between 18 and 55 years with a body weight ≥ 50 kg and body mass index 19–30 kg/m^2^. Participants had to be in good general health without clinically relevant medical illness, abnormal physical exam, electrocardiogram (ECG), or laboratory findings. Females had to have a negative pregnancy test and not be breastfeeding. Females of child-bearing potential had to agree to use highly effective contraception from the screening visit to until 40 days after the last study dose. For full inclusion and exclusion criteria, see the supplementary materials (Supplemental Methods S1).

### Procedures.

At the screening visit (day −28 to −2), a medical history was taken, demographics recorded, a physical examination performed, and eligibility criteria were assessed, including alcohol and drug screens, a HIV test and hepatitis panel, administration of the Beck Depression Inventory, a urine pregnancy test, a severe acute respiratory syndrome coronavirus 2 (SARS-CoV-2) test, and an ECG (Supplemental Methods S1). The schedule of postscreening assessments is shown in Supplemental Table S1.

Participants were confined to the clinical study unit in the morning of day −1. On day 1, infection was initiated with approximately 3,200 PfSPZ (NF54 strain; PfSPZ Challenge [Sanaria, Rockwell, MD]) by DVI, with participants discharged 2 hours postinoculation. Adverse events (AEs) and concomitant medications were monitored daily via phone call from day 2 until day 6. From day 7 until day 9, participants visited the clinical unit for daily assessments and were confined to the clinical unit from day 10. A 3-day course of antimalarial therapy with artemether-lumefantrine (20/120 mg) (Riamet^®^, Novartis, Basel, Switzerland) at the approved doses for treatment of acute uncomplicated malaria was initiated after the qPCR-defined target parasitemia of ≥ 5,000 parasites/mL was reached, or earlier if a participant had a malaria clinical score > 6 out of a maximum score of 42 (see below), or based on the investigator’s clinical discretion. Participants were discharged at least 72 hours after initiating antimalarial therapy once parasite clearance was achieved (see below) and they were asymptomatic. Three periods were therefore defined for analysis: the time from inoculation until parasitemia monitoring was started (days 1–6), the time from parasitemia monitoring (day 7) until artemether-lumefantrine administration (pretreatment), and the time from artemether-lumefantrine administration until parasite clearance (posttreatment).

Parasitemia level was determined by qPCR at the Institute of Tropical Medicine, Antwerp, Belgium, by a specific qPCR targeting the varATS (the acidic terminal segment in *Plasmodium falciparum* var genes) multigenic family (≈59 copies per genome), as previously described.^53^ Briefly, DNA was extracted from 200 μL of blood using the QIAamp 96 DNA blood kit (Qiagen, Germany), eluted in 200 μL of water, and 5 μL of DNA were used for qPCR analysis. The limit of detection was 50 parasites/mL of blood with results available within 4–8 hours of sampling. Parasite densities were obtained by interpolating cycle thresholds from a standard curve prepared with titrated samples containing known numbers of infected erythrocytes diluted in whole blood (10,000,000 to 1 parasites/mL). Parasite positivity was defined as ≥ 250 parasites/mL for a least one time point.^50^ Samples for parasite detection were obtained once daily on days 7–9, twice daily from day 10 until the target parasitemia of ≥ 5,000 parasites/mL was reached, before initiating antimalarial therapy, and at 2, 6, 8, 12, 16, 24, 36, 48, and 72 hours posttreatment to assess parasite clearance, once on the day of discharge, and once on day 28.

Malaria signs and symptoms were assessed using the malaria clinical score (Supplemental Methods S2).^50^ Adverse events consistent with malaria assessed using the malaria clinical score (myalgia, headache, arthralgia, fatigue/lethargy, malaise, chills/shivering/rigors, sweating/hot spells, anorexia, nausea, vomiting, abdominal discomfort, fever, tachycardia, and hypotension) were scored as 1 (mild), 2 (moderate), or 3 (severe), equating to Common Terminology Criteria for Adverse Events (CTCAE) grades 1, 2, and 3+, respectively. These AEs were classified as inoculum-related events and included in the malaria clinical score only if the participant was concurrently parasitemia positive. Assessments were conducted twice daily from day 10 until the day of discharge and once-daily at other time points (Supplemental Table S1).

Adverse events were monitored and coded according to the Medical Dictionary for Regulatory Activities (MedDRA) version 22.1, and vital signs and physical examination were performed throughout the study (Supplemental Table S1). Blood samples were taken for hematology, liver biochemistry, clinical chemistry, C-reactive protein (CRP), and coagulation assessments (Supplemental Table S1). Additionally, troponin T was assessed owing to prior reports of very rare cardiac events (idiopathic acute myocarditis/coronary syndrome) in PfSPZ by mosquito-bite studies.^36^^,^^54^^,^^55^ Twelve-lead ECGs were performed in triplicate at screening and on days 2 and 3 of antimalarial therapy.

### Endpoints.

Primary endpoints comprised safety/tolerability and parasite growth kinetics. Primary safety/tolerability endpoints were the incidence and severity of AEs considered related to PfSPZ Challenge by DVI; the change in malaria clinical score from inoculation until parasite clearance; changes from baseline in hematology, clinical chemistry and urinalysis parameters, vital signs, and ECG parameters. Primary endpoints characterizing blood-stage *P. falciparum* parasite growth were statistically derived and included time to first qPCR positivity (≥ 250 parasites/mL), parasitemia at first qPCR positivity, time to parasitemia of ≥ 5,000 parasites/mL, parasitemia at the first time of ≥ 5,000 parasites/mL, time to first dose of treatment with artemether-lumefantrine, the parasitemia at first dose of treatment with artemether-lumefantrine, and the number and proportion of participants with positive qPCR and parasitemia ≥ 5,000 parasites/mL between PfSPZ Challenge by DVI and day 28.

The incidence and severity of antimalarial treatment-related AEs was a secondary safety/tolerability endpoint. Additional secondary endpoints were the characterization of the blood-stage parasite profile using parasite growth rate expressed as the parasite multiplication rate (PMR) standardized to 48 hours and reported in log_10_ units (log_10_ PMR_48h_), and predicted time to reach the target parasitemia of ≥ 5,000 parasites/mL. Secondary pharmacodynamic endpoints defined the blood-stage clearance profile of artemether-lumefantrine, characterized by time to parasite clearance; log_10_ parasite reduction ratio per 48 hours (log_10_ PRR_48h_), that is, ratio of the parasite density at a specific time point to the parasite density 48 h later and expressed in log_10_; parasite clearance half-life (PC_50_), that is, the time taken for the parasite density to be reduced by 50% after the first dose administration of antimalarial therapy; and the time taken for the parasite density to be reduced by 99% after the first dose of antimalarial therapy (PC_99_).

### Statistical methods.

Statistical analysis was performed using SAS^®^ (SAS Institute Inc., Cary, NC; version 9.4). Baseline demographic data and the frequency of AEs were analyzed using descriptive statistics in all participants who were inoculated (safety population).

Outcomes for parasite growth kinetics and artemether-lumefantrine pharmacodynamics were analyzed for the pharmacodynamic population, including all inoculated participants with at least one available parasitemia level who received all artemether-lumefantrine doses and did not have protocol deviations that would have a relevant impact on outcomes.

Parasite growth kinetics were analyzed using descriptive statistics (geometric mean [GM], 95% CI), except time to parasitemia ≥ 5,000 parasites/mL, which was estimated using Kaplan–Meier time to event analysis (median, 95% CI). For the number and proportion of participants with positive qPCR and parasitemia ≥ 5,000 parasites/mL, corresponding two-sided exact 90% CIs were calculated (Clopper–Pearson).

To characterize the blood-stage parasite growth, a log-linear parasitemia growth model and an extended log-linear parasitemia growth model accounting for periodicity in the levels of qPCR-detectable parasitemia were evaluated based on the observed parasitemia data before artemether-lumefantrine administration (see Supplemental Methods S3 for details). The preferred model was chosen based on objective function value and common goodness of fit plots. The model parameters were estimated using Monolix v2019R1 (Antony, France: Lixoft SAS). Model-predicted parasitemia levels based on individual parameter estimates were assessed to derive model-based endpoints regarding parasite growth (i.e., PMR, log_10_ PMR_48h_, and time to reach 5,000 parasites/mL) using the log-linear mixed model extended to account for periodicity, as this showed a better fit to the individual parasitemia observations in terms of objective function value, as well as visual profile inspection (Supplemental Methods S3).

To assess artemether-lumefantrine pharmacodynamics, a log-linear model was fitted to the measured parasitemia data after artemether-lumefantrine administration. Parasite reduction and clearance parameters were then calculated from the estimates of the optimal linear regression model. Further details of the models are provided in the supplementary materials (Supplemental Methods S3). Additionally, for visualization purposes a Kaplan–Meier analysis was generated for the time to parasite clearance.

## RESULTS

### Participants.

Of the 90 volunteers screened as potential study participants, 63 did not fulfil the inclusion/exclusion criteria (Supplemental Table S2). Of the 27 eligible participants, 16 were enrolled in two sequential cohorts and 11 were unenrolled reserve participants. Enrolled participants comprised 10 males and 6 females, with a mean age of 42.4 years (range 22–54 years); all were of Caucasian self-declared ethnicity (Supplemental Table S3). All enrolled participants completed the study, received a full course of artemether-lumefantrine, and were included in the safety/tolerability and pharmacodynamic populations.

### Safety.

**Figure 2.**
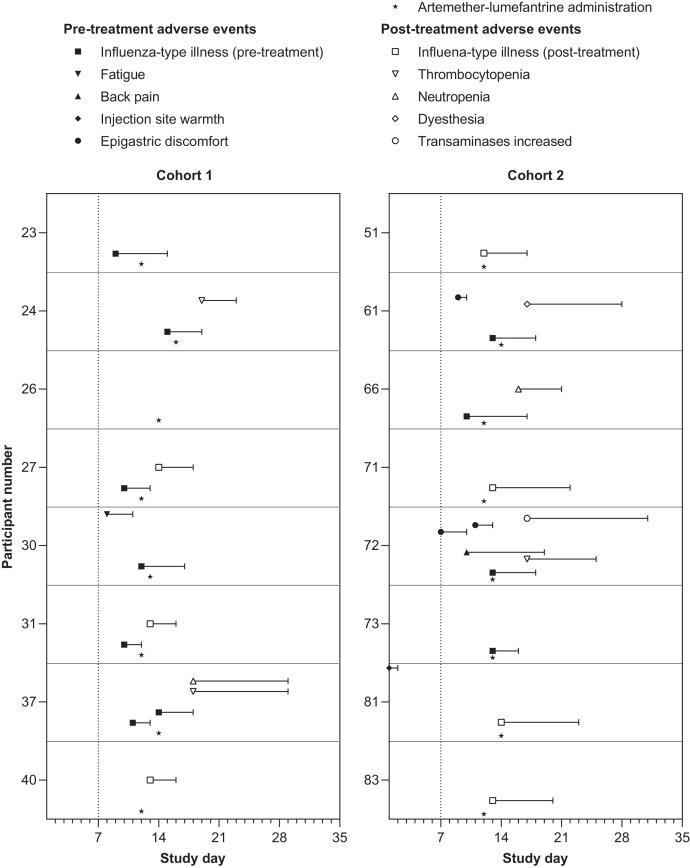
Frequency, duration, and timing of adverse events of any cause occurring throughout the study for individual participants. The dotted line at day 7 shows when parasitemia monitoring commenced.

There were no deaths, serious AEs or AEs leading to study withdrawal. A total of 31 AEs occurred in 15/16 participants across both cohorts (Figure 2). One AE of injection site warmth occurred before day 6 (Figure 2). After day 6 and before artemether-lumefantrine administration, 16 AEs were reported in 10 participants: 11 influenza-type illness, 3 epigastric discomfort, 1 fatigue, and 1 back pain (Figure 2). Following treatment, 14 AEs were noted in 12 participants: 7 influenza-type illness, 3 thrombocytopenia, 2 neutropenia, 1 dysesthesia, and 1 transaminases increased (Figure 2). Excluding the instance of injection site warmth, the GM time to any AE was 12.6 days (range 7–19 days; post-hoc analysis). Influenza-type illness lasted a GM of 4.3 days (range 2–9 days; post-hoc analysis), with symptoms consistent with malaria. While symptomatic, all participants tested negative for SARS-CoV-2. The case of dysesthesia occurred in the right thigh and was unrelated to the injection site.

Concomitant medication was given to alleviate malaria symptoms; 1/8 participants in cohort 2 received paracetamol both pre- and post-artemether-lumefantrine treatment. Following artemether-lumefantrine treatment, 6/8 participants received ibuprofen in cohort 1 and 8/8 in cohort 2, and 1/8 participants in each cohort received a single dose of domperidone (10 mg).

The majority of AEs (90.3% [29/31]) were grade 1 or 2 in severity. Two grade 3 AEs of neutropenia were reported in two participants (Table 1), which occurred following artemether-lumefantrine, though were not considered drug related, but related to malaria infection.

**Table 1:** Clinically relevant laboratory abnormalities

Participant ID	Cohort	Laboratory parameter, units	Value	Normal range	Onset	Duration	Adverse event (severity grade)
S024	1	Platelets, ×10^9^/mL	97	142–340	Day 19	4 days	Thrombocytopenia (grade 1)
S037	1	Platelets, ×10^9^/mL	79	142–340	Day 18	11 days	Thrombocytopenia (grade 1)
S037	1	Neutrophils, ×10^9^/mL	0.95	1.6–7.1	Day 18	11 days	Neutropenia (grade 3)
S066	2	Neutrophils, ×10^9^/mL	0.85	1.6–7.1	Day 16	5 days	Neutropenia (grade 3)
S072	2	ALT, U/L	141	≤41	Day 17	14 days	Transaminases increased (grade 2)
S072	2	AST, U/L	122	≤40	Day 17	14 days	
S072	2	Platelets, ×10^9^/mL	98	142–340	Day 17	8 days	Thrombocytopenia (grade 1)

ALT = alanine transaminase; AST = aspartate transaminase.

Laboratory abnormalities were most frequently observed following artemether-lumefantrine administration; increased alanine transaminase (ALT), aspartate transaminase, and lactate dehydrogenase (LDH) levels were only observed at this time. Seven laboratory abnormalities occurring in four participants were considered clinically relevant and recorded as AEs (Table 1). All of these laboratory abnormalities were considered related to malaria infection, and the occurrence of increased transaminases was also considered related to artemether-lumefantrine. There were no other drug-related AEs.

The overall incidence of laboratory abnormalities was comparable between the two cohorts (Supplemental Table S4). The most frequently observed laboratory abnormalities were high levels of CRP (93.8% [15/16]), ALT (62.5% [10/16]), and LDH (62.5% [10/16]), high ratios of monocytes/leukocytes (75.0% [12/16]) and reticulocytes/erythrocytes (68.8% [11/16]), and low levels of leukocytes (62.5% [10/16]) (Supplemental Table S4). All resolved spontaneously by the end of the study.

Changes in vital signs were generally small, except increased body temperature consistent with malaria in 13/16 (81.3%) participants (Supplemental Table S5). None of the other changes in vital signs were clinically relevant.

Based on ECG recordings, two participants in cohort 2 had increased heart rate. Two additional participants in cohort 2 had an increase in QT corrected using Bazett's formula (QTcB) from baseline of > 30 milliseconds and ≤ 60 milliseconds. One participant in cohort 1 showed negative T waves. No other ECG abnormalities, including abnormalities in QT corrected using Fridericia’s formula (QTcF) were observed (Supplemental Table S6). None of the observed ECG abnormalities were considered clinically relevant. There were no clinically relevant findings regarding cardiac troponin T.

### Malaria clinical score.

**Figure 3.**
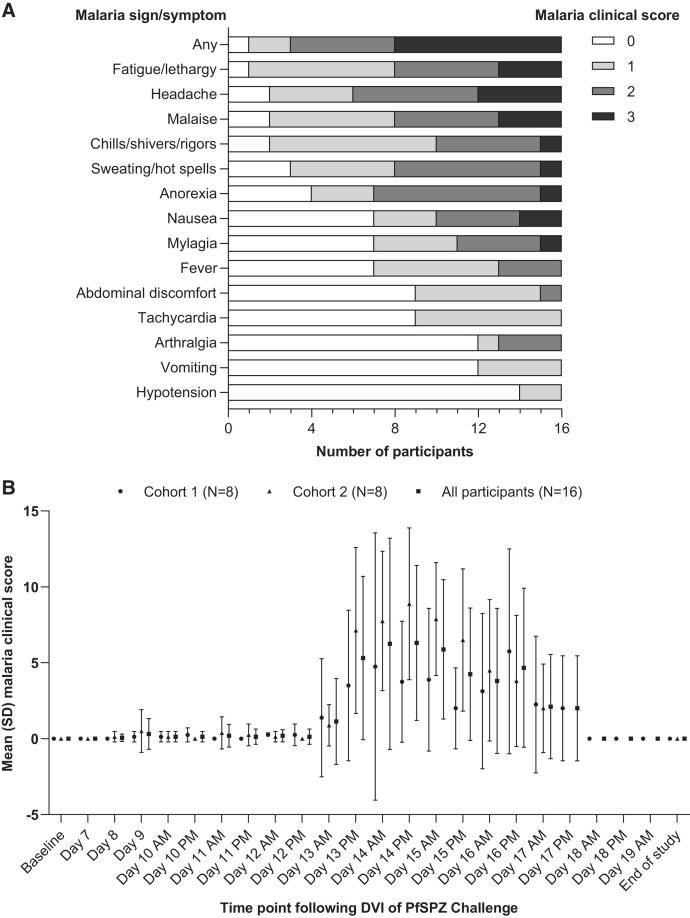
(**A**) Frequency of malaria clinical scores 0–3 for each sign and symptom. (**B**) Time course of the changes in mean malaria clinical score after direct venous inoculation (DVI) of PfSPZ Challenge. The malaria clinical score is specifically for recording signs and symptoms that are known to be associated with malaria. Note that malaria symptoms of clinical relevance were reported as adverse events mainly as “influenza-like” symptoms. Malaria severity grades corresponded to the Common Terminology Criteria for Adverse Events (CTCAE) grading scale grades 1–5 as follows: mild (1) equates to CTCAE grade 1, moderate (2) to CTCAE grade 2, and severe (3) to CTCAE grade 3 or above.

Malaria signs/symptoms were noted in 15/16 participants, most commonly fatigue/lethargy, with 8/16 having a score of 3 (severe) for any individual sign/symptom (Figure 3A). Positive malaria clinical scores were reported as early as day 8 post-inoculation but had resolved in all participants by day 18 (Figure 3B). The maximum malaria clinical score was 24 on the morning of day 14 post-inoculation and 13/16 participants had malaria clinical scores > 6. The mean highest malaria clinical score was 2.0 (SD 3.1) following inoculation before artemether-lumefantrine administration, but increased to 11.3 (SD 5.7) post-treatment.

### Parasite growth kinetics.

**Figure 4.**
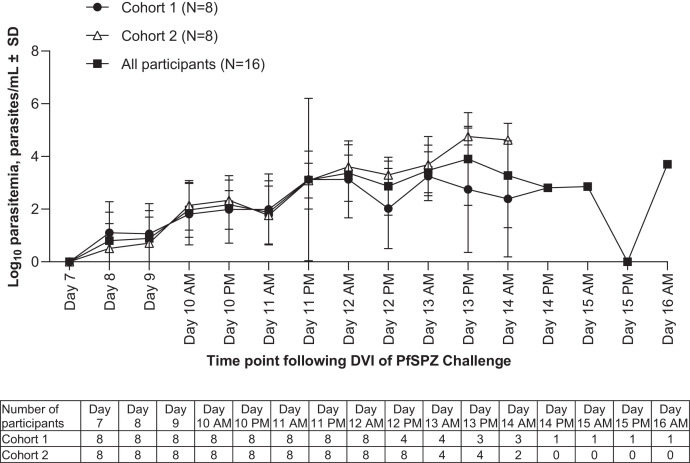
Mean log_10_ parasitemia from direct venous inoculation (DVI) of PfSPZ Challenge until artemether-lumefantrine administration. The number of evaluable participants at each time point is shown in the table.

All 16 inoculated participants developed parasitemia following PfSPZ Challenge by DVI, and all had parasitemia exceeding the qPCR-defined target of ≥ 5,000 parasites/mL. The time course of parasitemia before artemether-lumefantrine administration is shown in Figure 4, with artemether-lumefantrine initiated between Day 13 AM and Day 16 PM.

**Figure 5.**
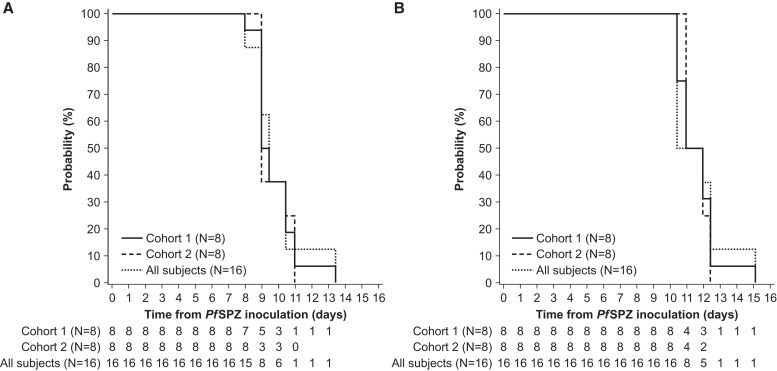
Kaplan–Meier estimates. (**A**) Time to first parasitemia. (**B**) Time to reach quantitative polymerase chain reaction (qPCR)-defined parasitemia target of ≥ 5,000 parasites/mL.

Primary endpoints characterizing blood-stage *P. falciparum* parasite growth are shown in Table 2. The GM time to parasitemia was 9.7 days (95% CI 9.1–10.4), with a parasitemia level at the first positive qPCR result of GM 511 (95% CI 369–709) parasites/mL. The Kaplan–Meier estimate of median time to parasitemia ≥ 5,000 parasites/mL was 11.5 days (95% CI 10.4–12.4) (Table 2, Figure 5).

**Table 2:** Primary pharmacodynamic endpoints characterizing parasite growth

Pharmacodynamic endpoint	Cohort 1 (*N* = 8)	Cohort 2 (*N* = 8)	All participants (*N* = 16)
Time to first qPCR parasite positivity, days			
Geometric mean (two-sided 95% CI)	9.8 (8.6–11.1)	9.6 (8.9–10.4)	9.7 (9.1–10.4)
Geometric SD	1.17	1.10	1.13
Min; max	8.0; 13.4	9.0; 11.0	8.0; 13.4
Parasitemia at first positive qPCR, parasites/mL			
Geometric mean (two-sided 95% CI)	367 (280–482)	712 (406–1,249)	511 (369–709)
Geometric SD	1.4	2.0	1.8
Min; max	258; 642	266; 1,850	258; 1,850
Time to parasitemia ≥ 5,000 parasites/mL, days			
Median (95% CI)	11.2 (10.4–12.4)	11.5 (11.0–12.4)	11.5 (10.4–12.4)
25th quantile (95% CI)	10.4 (10.4–12.0)	11.0 (11.0–12.0)	10.7 (10.4–11.0)
75th quantile (95% CI)	12.4 (10.4–15.1)	12.2 (11.0–12.4)	12.4 (11.0–12.4)
Parasitemia at first time of ≥ 5,000, parasites/mL			
Geometric mean (two-sided 95% CI)	12,807 (7,736–21,203)	18,831 (8,739–40,579)	15,530 (10,268–23,488)
Geometric SD	1.8	2.5	2.2
Min; max	5,890; 31,644	6,498; 76,133	5,890; 76,133
Time to first AL dose, days			
Geometric mean (two-sided 95% CI)	12.1 (11.0–13.3)	12.0 (11.5–12.7)	12.1 (11.5–12.7)
Geometric SD	1.1	1.1	1.1
Min; max	11.0; 15.0	11.4; 13.0	11.0; 15.0
Parasitemia after the first AL dose, parasites/mL*			
Geometric mean (two-sided 95% CI)	3,937 (219–70,890)	9,454 (3,662–24,409)	6,101 (1,587–23,450)
Geometric SD	31.7	3.1	12.5
Min; max	1; 55,519	1,945; 52,254	1; 55,519

AL = artemether-lumefantrine; qPCR = quantitative polymerase chain reaction.

*Values were computed using the first available assessment after the first dose of artemether-lumefantrine (after 2 hours); there was no assessment taken at the time of artemether-lumefantrine administration (*t* = 0).

Target parasitemia was achieved around 43 hours after first qPCR positive parasitemia, at a GM parasitemia level of 15,530 (95% CI 10,268–23,488). Artemether-lumefantrine administration was triggered in 15/16 participants by the target parasitemia being met, and in the remaining participant by a malaria clinical score > 6, though their parasitemia level was close to the threshold (4,977 parasites/mL). Overall, GM parasitemia levels were 6,101 parasites/mL (95% CI 1,587–23,450) at treatment initiation. The GM time to artemether-lumefantrine administration was 12.1 days (95% CI 11.5–12.7). The mean parasitemia at artemether-lumefantrine initiation was lower than the parasitemia level at the time artemether-lumefantrine administration was triggered (15,530 parasites/mL) because the parasite cycle causes the parasite density to fluctuate every 48 hours following sequestration/release. Thus, treatment was only given when laboratory reported a qPCR determined parasite density of ≥ 5,000 parasites/mL, (4–8 hours after that the sample was taken); by the time that the clinic gave the treatment parasite density in blood had cyclically gone down.

The parasite growth rate indicated a log_10_ PMR_48h_ of 1.3 (95% CI 1.2–1.3), and the predicted time from positive parasitemia to reaching the target parasitemia of ≥ 5,000 parasites/mL was approximately 49 hours (Table 3).

**Table 3:** Model-derived estimates of parasite growth after direct venous inoculation of PfSPZ Challenge

Parasite growth estimate	Cohort 1 (*N* = 8)	Cohort 2 (*N* = 8)	All participants (*N* = 16)
Log_10_ PMR_48h_			
Mean (two-sided 95% CI)	1.2 (1.2–1.3)	1.3 (1.3–1.3)	1.3 (1.2–1.3)
Min; max	1.2; 1.3	1.2; 1.3	1.1; 1.3
Predicted time to positive parasitemia, hours			
Mean (two-sided 95% CI)	225.6 (196.5–259.1)	221.5 (205.6–238.6)	223.5 (208.7–239.4)
Min; max	196; 316	205; 249	196; 316
Predicted time to ≥ 5,000 parasites/mL, hours			
Mean (two-sided 95% CI)	275.1 (243.1–311.4)	270.5 (255.1–287.0)	272.8 (257.0–289.7)
Min; max	241; 361	251; 294	241; 361

PfSPZ = *Plasmodium falciparum* sporozoite; PMR = parasite multiplication rate.

### Artemether-lumefantrine pharmacodynamics.

**Figure 6.**
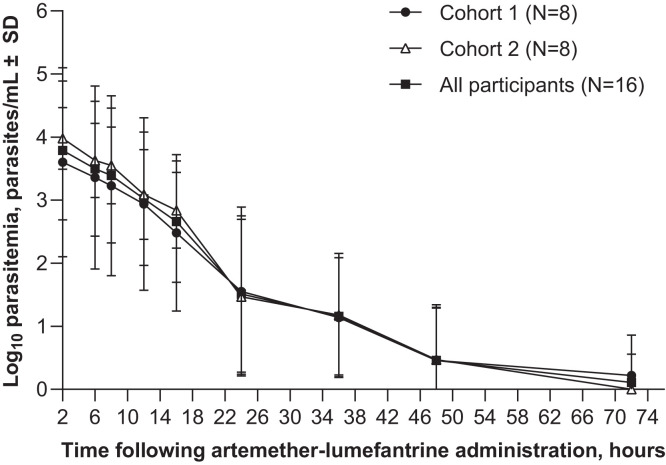
Mean log_10_ parasitemia after administration of the first artemether-lumefantrine dose.

**Figure 7.**
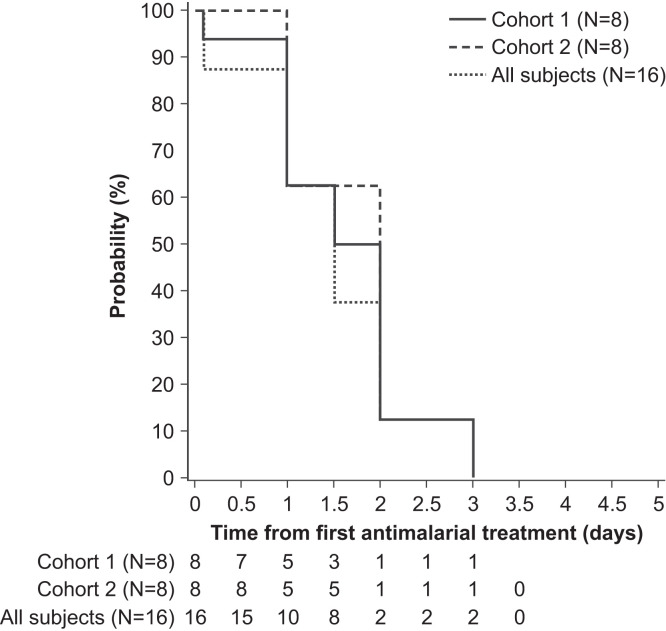
Kaplan–Meier estimates of time to parasite clearance after commencement of artemether-lumefantrine treatment.

Artemether-lumefantrine was associated with a rapid decline in parasitemia (Figure 6). The GM time to parasite clearance was 1.3 days (95% CI 0.9–2.1). All subjects had parasite clearance by day 3 (Figure 7). The mean log_10_ PRR_48h_ was 3.6 (95% CI 3.4–3.7) with a PC_50_ of 4.1 hours (95% CI 3.9–4.3), and a PC_99_ of 27.0 hours (95% CI 25.7–28.4) (Table 4).

**Table 4:** Model-derived parasitemia clearance parameters after commencing artemether-lumefantrine antimalarial therapy

Parasite clearance parameter	Cohort 1 (*N* = 8)	Cohort 2 (*N* = 8)	All participants (*N* = 16)
Log_10_ PRR_48h_	3.9 (3.7–4.1)	3.2 (2.9–3.4)	3.6 (3.4–3.7)
PC_50_, hours	3.7 (3.5–4.0)	4.5 (4.2–5.0)	4.1 (3.9–4.3)
PC_99_, hours	24.7 (23.2–26.3)	30.2 (27.9–32.9)	27.0 (24.7–28.4)

PC_50_ = parasite clearance half-life; PC_99_ = time to reach parasite clearance of 99%; PRR = parasite reduction ratio. Parasite clearance is geometric mean (95% CI), other values are mean (95% CI) estimated using the inverse-variance method to calculate the weighted average linear regression slope.

## DISCUSSION

Controlled human malaria infection using PfSPZ Challenge by DVI has the potential to expand the currently limited capability for evaluating new chemical entities with blood-stage antimalarial activity. Supported by a sound scientific rationale and extensive published literature,^4^^,^^16^^–^^29^^,^^47^^–^^51^ this prospective methodological study demonstrated the safety, tolerability, and feasibility of the PfSPZ Challenge by DVI for evaluating antimalarial drug candidates with blood-stage activity. In particular, this is the first time that the parasite clearance curve following artemether-lumefantrine treatment has been this closely defined in the CHMI setting (nine time points after treatment over the first 3 days).

Safety/tolerability findings were of an acceptable frequency, severity, and duration, and similar to published CHMI trials using PfSPZ Challenge by DVI.^16^^–^^30^^,^^47^^–^^51^ Excepting injection site reactions, reports of AEs over the first 7 days following DVI of PfSPZ Challenge are uncommon.^16^^–^^29^^,^^47^^–^^51^ In the current study, there was only one AE reported before day 7 (injection site warmth). Similar to other studies in malaria-naïve volunteers,^16^^–^^20^^,^^28^^,^^47^^–^^49^ the majority of AEs occurred after parasitemia was established, were consistent with the symptoms of malaria, and resolved following parasite clearance. There were some differences in AEs between the two study cohorts, with fever, increased heart rate, and QTcB prolongation only reported in cohort 2. Parasite densities were also higher in cohort 2, with slightly slower parasite clearance and this may have led to the different AE profiles. However, these variations are most likely a result of variability between participants for this small sample size.

Comparable to previous studies of PfSPZ Challenge by DVI, most AEs were grade 1 or 2.^16^^,^^18^^–^^20^^,^^22^^,^^48^^–^^50^ There were two grade 3 AEs of neutropenia. Neutropenia has been reported previously in CHMI with PfSPZ Challenge by DVI,^16^^,^^22^^,^^28^^,^^47^^,^^48^ as well as other CHMI models.^10^^,^^32^^,^^37^^,^^38^^,^^42^ In this case, neutropenia is thought represent a shift in the granulocyte balance towards the marginated pool, that is, the prolonged transit of cells through organs (liver, spleen, bone marrow) which results in an apparent decrease in circulating neutrophils.^56^^,^^57^ Transient thrombocytopenia and asymptomatic increases in hepatic transaminases were also observed here, in other studies following PfSPZ Challenge by DVI,^16^^,^^22^^,^^29^^,^^47^^,^^48^^,^^58^ and other CHMI models.^10^^,^^15^^,^^32^^,^^34^^,^^37^^–^^39^^,^^42^^,^^58^^,^^59^ The pathophysiology of transient thrombocytopenia is hypothesized to result from decreased platelet survival following platelet activation, mediated by adenosine diphosphate released during erythrocyte hemolysis.^37^ Transient hepatic transaminases elevations appear to be more common at higher parasitemia levels.^58^^,^^59^ This could be explained by an acute inflammatory response accompanied by oxidative stress in malaria-naïve healthy volunteers.^58^^,^^59^

Consistent with previous studies in non-immune volunteers using an inoculum of 3,200 PfSPZ Challenge,^19^^,^^20^^,^^22^^,^^26^^,^^28^^,^^47^^–^^50^ all 16 participants in this study developed parasitemia. Previous studies using this CMHI model and using qPCR for parasite assessment have reported a median time to parasitemia of 9 days,^28^^,^^49^ mean of 9.2 days,^17^ or GM between 10.6 and 13.8 days.^16^^,^^47^^,^^48^^,^^50^ The prepatent period in the current study was similar with a GM of 9.7 days (range 8.0–13.4). For comparison, for infection established with PfSPZ by mosquito bite, the pre-patent period ranges from 6 to 23 days, but is most commonly around 7–12 days.^7^^,^^15^^,^^30^^,^^35^^,^^37^^,^^39^^,^^43^

The estimated parasite growth rate (log_10_ PMR_48h_ of 1.3 [95% CI 1.2–1.3]) observed in our study was consistent with estimates from published data from CHMI studies using PfSPZ Challenge (1.1 [95% CI 0.93–1.3]),^33^ slightly lower than CHMI using pRBCs (1.5 [95% CI 1.4–1.5]), but higher than observed for mosquito-bite studies with *P. falciparum* 3D7 (0.9 [95% CI 0.86–1.0]),^33^ or *P. falciparum* NF54 (1.0 [95% CI 0.9–1.1]).^33^ With PfSPZ Challenge by DVI, although each individual participant shows parasite cycle synchronicity similar to studies using pRBCs, the time at which parasites are released into the blood varies between individuals; hence, across a cohort the synchronicity is not seen clearly. To fully characterize parasite growth following PfSPZ Challenge by DVI, the time taken for PfSPZ to reach the blood needs to be known. In this study, the sample size was too small to estimate this parameter, but data could be amalgamated across several similar studies to do this, as was the case for studies using pRBCs.

Artemether-lumefantrine was used in this exploratory study as a registered rescue medication due to its well described antimalarial efficacy and safety in CHMI models in malaria-naïve volunteers.^48^ The parasite clearance half-life observed in this study with artemether-lumefantrine of 4.1 hours (95% CI 3.9–4.3) was similar to that reported in volunteers with malaria parasitemia established via the intravenous administration of pRBCs for the candidate blood-stage antimalarial drugs SJ733 (3.6 hours),^10^ and artefenomel (3.6 hours),^8^ and was faster than for the candidate antimalarial DSM265 (9.4 hours),^9^ and the approved antimalarial mefloquine (6.2 hours).^9^ Thus, although requiring verification, we are confident that PfSPZ Challenge by DVI would be able to discern acceptable blood-stage efficacy for investigational molecules relative to artemether-lumefantrine.

In consideration of participant safety, artemether-lumefantrine administration was triggered either by the target parasitemia of ≥ 5,000 parasites/mL blood determined by qPCR, by a clinical malaria score > 6, or at the investigator’s discretion.^59^ In studies using pRBCs to establish malaria infection, > 1,000 parasites/mL blood has been sufficient to demonstrate blood-stage antimalarial efficacy.^8^^,^^9^^,^^32^ In one such study, the log_10_ PRR with artemether-lumefantrine was 2.9 (95% CI 2.1–3.7) in volunteers with a median parasitemia of 2,926 parasites/mL (range 1,501–8,524).^32^ In our study, GM parasitemia at the time of treatment initiation was 6,101 parasites/mL blood (range 1–55,519) and the log_10_ PRR_48h_ for artemether-lumefantrine was 3.6 (95% CI 3.4–3.7). Thus, there may be some scope to further reduce the parasite threshold at which treatment is initiated, while still allowing characterization of parasite clearance kinetics. However, these data provide reassurance of the feasibility of reaching adequate parasitemia levels to support pharmacodynamic analysis of future drug candidates, while achieving a reasonable control of malaria symptoms.

Timely evaluation of parasitemia using qPCR limits participants’ risk from malaria symptoms compared with microscopic parasite assessments.^16^^,^^21^ In this study, we obtained qPCR samples twice daily, both to minimize the frequency and severity of AEs by rapidly initiating artemether-lumefantrine once the target parasitemia was reached, and to provide the high density of data points required to develop a pharmacodynamic model for the evaluation of blood-stage antimalarial activity (to be reported separately). However, it may not be necessary to conduct such frequent sampling in future studies.^16^^,^^21^ Note that the limit of detection was 50 parasites/mL of blood in this study, and a more sensitive method would allow earlier detection of parasitemia and potentially permit a lower target parasitemia to be used.^16^^,^^18^

Our study has some key limitations. It is exploratory, with a relatively small sample size, providing supportive rather than confirmatory evidence of safety/tolerability and feasibility of the PfSPZ Challenge by DVI as a model suitable for the investigation of blood-stage malaria activity. Our study solely evaluated a fully curative dose of an approved malaria drug and we are not able to directly compare our findings with other CHMI models where new chemical entities are also tested at sub-therapeutic doses. Also, our results cannot necessarily be directly compared with other CHMI studies which use different PfSPZ Challenge strains or parasite clones. Although the 3,200 PfSPZ Challenge dose appears suitable for the evaluation of blood-stage drug efficacy in malaria-naïve volunteers, it may not be optimal in semi-immune African populations.^26^ Finally, it should be noted that the malaria clinical score is not a validated tool and was used as an additional method of limiting patient discomfort by triggering antimalarial therapy at a low level of mild symptoms, regardless of parasitemia levels.

The establishment of the PfSPZ Challenge by DVI as a CHMI model for evaluating new antimalarial drugs with blood-stage activity would provide a valuable alternative to CHMI studies that use PfSPZ transmitted via mosquito bites or intravenous administration of pRBCs to initiate *P. falciparum* infection. Importantly, it would enable additional sites to conduct these studies, accelerating the development of new antimalarial therapies.

## Supplemental files


Supplemental materials
