# Supplementary material for: Safety, Tolerability, and Parasite Clearance Kinetics in Controlled Human Malaria Infection after Direct Venous Inoculation of Plasmodium falciparum Sporozoites: A Model for Evaluating New Blood-Stage Antimalarial Drugs
Source: Am J Trop Med Hyg. 2022 Aug 29;107(4):804–14. doi: 10.4269/ajtmh.21-1297 (PMC9651526; doi:10.4269/ajtmh.21-1297)
Supplement: Supplementary file 1 [file tpmd211297.SD1.pdf]

Supplemental Materials

Supplemental Methods S1: Eligibility criteria.

Inclusion criteria:

1. Informed Consent Form signed voluntarily before any study-related procedure is performed, indicating that the participant understands the purpose of and procedures required for the study and is willing to participate in the study, including administration of registered antimalarial therapy;
2. Male or female, between 18 and 55 years old (extremes included) at screening;
3. Body weight of at least 50 kg and a body mass index (BMI) of 19.0 to 30.0 kg/m^2 (extremes included);
4. Good general health without clinically relevant medical illness, physical exam findings including vital signs, and laboratory abnormalities (e.g., without liver transaminases >1x the upper limit of normal [ULN] and as defined in the protocol) as determined by the Investigator;
5. Willing to adhere to the prohibitions and restrictions specified in this protocol, including willingness to stay confined to the inpatient unit for the required duration and willingness to avoid travelling outside of Benelux during the study period;
6. Female participants should fulfil one of the following criteria:
a. At least 1 year postmenopausal (amenorrhea >12 months and follicle stimulating hormone [FSH] >30 mIU/mL) prior to screening;
b. Surgically sterile (bilateral oophorectomy, hysterectomy or bilateral salpingectomy);
c. Will use contraceptives as outlined in inclusion criterion 7;
7. Female participants of childbearing potential (excluding females with female partners) must agree to the use of a highly effective method of birth control from the screening visit until 40 days after the EOS visit at Day 28 (covering a full menstrual cycle of 30 days starting after 5 half-lives of last dose of Riamet®); Note: Highly effective birth control methods include: combined (estrogen- and progestogen-containing) oral/intravaginal/transdermal hormonal contraception associated with inhibition of ovulation, progestogen-only oral/injectable/implantable hormonal contraception associated with inhibition of ovulation, intrauterine device, intrauterine hormone-releasing system, bilateral tubal occlusion, vasectomized partner or sexual abstinence from heterosexual intercourse.
8. Female participant has a negative pregnancy test at screening and upon admission in the clinical unit; Note: Pregnancy testing will consist of serum β-human chorionic gonadotropin (β-HCG) tests at screening and at the EOS visit and a urine β-HCG tests on Day -1, in all women.
9. Different ways of being reachable 24/7 (e.g., by mobile phone, regular phone or electronic mail) during the whole study period.

Exclusion criteria:

1. Nursing (lactating) women;
2. Participation in any other clinical drug or vaccine study within 30 days (or 5 half-lives for drugs) preceding the day of *Pf*SPZ-DVI Challenge (whichever is longer), or plans to participate in other investigational drug or vaccine research during the study period;
3. Participants who took standard vaccinations within 3 months before the start of the study or are planning to take standard vaccinations during the study period up to 8 weeks after *Pf*SPZ-DVI Challenge;
4. Blood product donation to any blood bank during the 8 weeks (whole blood) or 4 weeks (plasma and platelets) prior to admission in the clinical unit on Day -1;
5. Mean ECG outside normal range and deemed clinically relevant by the Investigator. Examples of clinically significant ECG abnormalities for this study include:
- PR-interval >220 ms;
- QRS-complex >120 ms;
- Absolute QT greater than >500 ms;

- QT interval corrected according to Bazett's formula (QTcB) or QT interval corrected according to Fridericia's formula (QTcF >450 ms for male participants, >470 ms for female participants;
- Pathologic Q wave;
- Significant ST-T wave changes;
- Left or right ventricular hypertrophy;
- Non-sinus rhythm except isolated premature atrial contractions and ventricular extrasystole <2 per 10 s ECG lead;
- Incomplete left bundle branch block, or complete or intermittent right or left bundle branch block;
- Second or third degree A-V heart block.

6. Seropositive human immunodeficiency virus (HIV), hepatitis A immunoglobulin M (IgM) antibody, hepatitis B virus (HBV) (hepatitis B surface antigen [HBsAg]), hepatitis C virus (HCV) (antibody), hepatitis D antibody, hepatitis E IgM antibody, cytomegalovirus (CMV) IgM antibody or Epstein Barr Virus (EBV) IgM antibody;
7. Previous or current diagnosis of hepatitis including but not limited to viral hepatitis, auto-immune hepatitis, non-alcoholic steatohepatitis (NASH), alpha-1-antitrypsin deficiency, alcoholic liver disease, primary biliary cholangitis (PBC), primary sclerosing cholangitis (PSC), hemochromatosis, Wilson disease or suspected hepatocellular carcinoma (HCC).
8. History or presence of diagnosed food or known drug allergies (including but not limited to allergy to any of the antimalarial medications to be used in the study), or history of anaphylaxis or other severe allergic reactions; Note: Participants with seasonal allergies/hay fever, house dust mite or allergy to animals that are untreated and asymptomatic at the time of dosing can be enrolled in the study.
9. History of convulsion or severe head trauma, excluding fever convulsion under 5 years of age; Note: A medical history of a single febrile convulsion during childhood is not an exclusion criterion.
10. History of serious psychiatric condition that may affect participation in the study or preclude compliance with the protocol, including but not limited to past or present psychoses, disorders requiring lithium, a history of attempted or planned suicide, more than one previous episode of major depression, any previous single episode of major depression lasting for or requiring treatment for more than 6 months, or any episode of major depression during the 5 years preceding screening; Note: The Beck Depression Inventory will be used as an objective tool for the assessment of depression at screening. In addition to the conditions listed above, participants with a score of 20 or more on the Beck Depression Inventory and/or a response of 1, 2 or 3 for item 9 of this inventory (related to suicidal ideation) will not be eligible for participation. Participants with a Beck score of 17 to 19 may be enrolled at the discretion of the Investigator if they do not have a history of the psychiatric conditions mentioned in this criterion and their mental state is not considered to pose additional risk to the health of the volunteer or to the execution of the study and interpretation of the data gathered.
11. A medical, occupational or family problem as a result of alcohol or illicit drug abuse during the past 12 months or current alcohol or illicit drug abuse or addiction (positive alcohol breath test or positive drug screen for amphetamines, barbiturates, benzodiazepines, cannabinoids, cocaine or opiates at screening or upon check-in at the clinical unit); Note: Excessive use of alcohol is defined as an intake of >21 units per week for males and >14 units per week for females where one alcohol unit is defined as 10 mL or 8 g of pure alcohol. A single unit is equal to one 25-mL (single) measure of whisky (alcohol by volume [ABV] 40%), or a third of a pint of beer (190 mL; ABV 5-6%) or half a standard (175 mL) glass of wine (ABV 12%).
12. Participants are non-smokers or ex-smokers for more than 90 days prior to screening, or smoke no more than 5 cigarettes per day. If users of nicotine products (i.e., spray, patch, e-cigarette, etc.), they should use the equivalent of no more than 5 cigarettes per day. Participants must agree to abstain from smoking while in the unit;
13. Use of any prescription drugs, herbal supplements (e.g., St John's Wort) or over-the-counter medication within 7 days or 5 half-lives (whichever is longer) prior to the *Pf*SPZ-DVI Challenge, or an

anticipated requirement for the use of these during the course of the study; Note: If necessary, the incidental use of non-steroidal anti-inflammatory drugs (NSAIDs) and paracetamol (2 g/day, 10 g/week) may be acceptable at the Investigator's discretion and will be documented in the eSource system. The use of nutritional supplements during this time that are not believed to have the potential to affect participant safety nor the overall results of the study, may be permitted on a case-by-case basis by the Investigator.
14. Any surgical or medical condition possibly affecting drug absorption (e.g., cholecystectomy, gastrectomy, bowel disease), distribution, metabolism or excretion;
15. Personnel (e.g., Investigator, sub-investigator, research assistant, pharmacist, study coordinator or anyone mentioned in the delegation log) directly involved in the conduct of the study;
16. Any condition that in the opinion of the Investigator would jeopardize the safety or rights of a person participating in the study or would render the person unable to comply with the protocol;
17. Personal history of malaria;
18. Volunteer has travelled to or lived in a malaria-endemic area within 6 months prior to planned study enrolment;
19. Plans to travel to malaria-endemic region during the study period up to last follow-up visit;
20. Previous participation in any malaria vaccine or Controlled Human Malaria Infection (CHMI) study/VIS;
21. Falling in moderate or higher risk category for a fatal or non-fatal cardiovascular event within 5 years (> 5%) determined by a validated risk estimation system, e.g., SCORE;
22. Use of systemic antibiotics with known antimalarial activity within 5 half-lives of *Pf*SPZ-DVI Challenge (e.g., trimethoprim-sulfamethoxazole, doxycycline, tetracycline, clindamycin, erythromycin, fluoroquinolones or azithromycin) or an anticipated requirement for the use of these during the study period;
23. Receipt of blood or blood-derived products (including immunoglobulin) within 3 months prior to screening. Receipt of packed red blood cells (RBCs) given for an emergent indication in an otherwise healthy person, and not required as ongoing treatment is not exclusionary (for example packed RBCs emergently given during an elective surgery).
24. Participants who test positive for severe acute respiratory syndrome coronavirus 2 (SARS-CoV-2).

Note: In case of an out-of-range clinical laboratory test (as defined in the protocol), vital sign or ECG value that will determine a participant's eligibility, or in case of a positive drug screen, a retest or expert evaluation can be requested. Results of any retest must be available prior to inoculation. The result of the retest will be considered for participant eligibility at the Investigator's discretion. Participants can be rescreened at the discretion of the Investigator.

Supplemental Table S1: Post screening assessment schedule.

Assessment	Study day/visit								
	Day −1	Day 1	Day 2–6	Day 7–9	Day 10	Parasite positive[a]	Parasitemia threshold[b]	Parasite clearance[c]	Day 28
Urine pregnancy test	●								●
Drug and alcohol screen	●								
SARS-Cov-2 test				●					
Height and weight	●								●
Physical examination	●				●	●	●	●	●
Vital signs	●	●			●	●	●	●	●
Parasitemia (qPCR)		●		●	●	●	●	●	●
Malaria clinical score		●		●	●	●	●	●	●
Adverse events	●	●	●	●	●	●	●	●	●
Concomitant medication	●	●	●	●	●	●	●	●	●
Hematology/biochemistry/urinalysis[d]	●			●	●	●	●	●	●
CRP and coagulation parameters[e]	●			●	●	●	●	●	●
12-lead ECG							●		

CRP, C-reactive protein; ECG, electrocardiograph; qPCR, quantitative polymerase chain reaction; SARS-CoV-2, severe acute respiratory syndrome coronavirus 2.

[a] Day of first PCR ≥250 parasites/mL until day of first PCR ≥5,000 parasites/mL.

[b] Day of first PCR ≥5,000 parasites/mL until ≥72 hours after initiating artemether-lumefantrine therapy.

[c] Day of parasite clearance (PCR 0 parasites/mL) and at least 72 hours after initiating artemether-lumefantrine therapy; day of discharge from clinical unit.

[d] Liver biochemistry was conducted at all indicated visits and biochemistry other than liver and urinalysis on day −1, day 10, day of parasite positivity and end of study. Liver biochemistry: albumin, aspartate aminotransferase, alanine aminotransferase, alkaline phosphatase, gamma-glutamyl transferase, total and direct bilirubin and total serum proteins; Biochemistry other than liver: sodium, potassium, chloride, bicarbonate, urate, inorganic phosphate, creatinine, C-reactive protein; estimated glomerular filtration rate, glucose, lactate dehydrogenase, blood urea nitrogen and creatine phosphokinase; Hematology: hemoglobin, hematocrit, RBC count, mean corpuscular hemoglobin, mean corpuscular hemoglobin concentration, mean corpuscular volume, white blood cell count, platelet count, reticulocytes, neutrophils, eosinophils, basophils, lymphocytes and monocyte; Urinalysis: glucose, protein, nitrite, pH and occult blood; Other: High sensitive cardiac troponin T.

[e] Coagulation: prothrombin time and activated partial thromboplastin time (at screening and on day −1 only), and international normalized ratio.

Supplemental Methods S2: Malaria clinical score.

Grading of the signs and symptoms specified in the table beneath was performed according to the Common Terminology Criteria for Adverse Events (CTCAE) version 5.0 grading scale at all relevant protocol-specified time points, during the previous 24 hours (continuously) if once daily assessments were required or during the previous 12 hours (continuously) if assessments were performed twice daily, in accordance with the following:

- Absent = 0
- Mild = 1; equates to CTCAE grade 1
- Moderate = 2; equates to CTCAE grade 2
- Severe = 3; equates to CTCAE grade 3 or above

**Participant ID:**	**Date:**	**Time of Onset:**<br>**Stop:**
**Symptom/Sign**	**Score (0 to 3)**	**qPCR**
Headache		☐ positive
Myalgia (muscle ache)		☐ negative
Arthralgia (joint ache)		
Fatigue/lethargy		
Malaise (general discomfort/uneasiness)		
Chills/Shivering/Rigors		
Sweating/hot spells		
Anorexia		
Nausea		
Vomiting		
Abdominal discomfort		
Fever		
Tachycardia		
Hypotension		
**TOTAL SCORE**	**…/42**	

Supplemental Methods S3: Pharmacodynamic modelling.

Blood-stage parasite profile before antimalarial drug administration

To characterize the blood-stage parasite profile before treatment with artemether-lumefantrine, first, a log-linear mixed effect model was fitted to the measured parasitemia (in count/mL, transformed on natural log scale) data prior to artemether-lumefantrine administration. In this model, for each individual i:

$$\begin{cases} \dfrac{d\ log\, Parasitaemia_i}{dt} = GR_i \\ logParasitaemia_{0,i} = logF_{inoc,i} + logP_{inoc} - logV_{blood} \end{cases}$$

where t is the time in hours since inoculation, GR_i is the individual parasitaemia growth rate (measured in 1/hour), $Parasitaemia_{0,i}$ is the individual parasitemia at time of inoculation ($t = 0$), $F_{inoc,\,i}$ is the individual inoculum viability, P_{inoc} is the inoculum size, which is fixed to 3,200 parasites, and V_{blood} is the blood volume (in mL units) which is fixed to 5,000 mL for all individuals:

If the measured parasitaemia profile showed a cyclic behavior due to synchronicity of the parasites and their sequestration during the late stages of their lifecycle, an attempt will be made to model this behavior using the following extended version of the log-linear model:

$$\begin{cases} \dfrac{d\ \ log\, Parasitaemia_i}{dt} = GR_i \\ log\ Parasitaemia_{0,i} = logF_{inoc,i} + logP_{inoc} - logV_{blood} \\ logParasitaemia_{visible,i} = log\ Parasitaemia_i + log\left\{1 - 0.5F_{max,i}\left[1 - \cos\left(\dfrac{2\pi(t/24 - \mathrm{D}_{t,i})}{P_{len,i}}\right)\right]\right\} \end{cases}$$

where $Parasitaemiavisible,i$ denotes the measured parasitemia, $\mathrm{D}t,i$ = $\phi iPlen,i$, $Fmax,i$ denotes the individual maximum fraction of hidden parasites, $Plen,i$ denotes the individual period length of the oscillation (in hours), and ϕi denotes the individual oscillation phase.

Parameter estimation was performed using Monolix (Antony, France: Lixoft SAS). By default, all of the model parameters described above were estimated, including their inter-individual variability (IIV) distributions. If, due to limited data size, the relative standard error (RSE) of the estimates exceeded 40%, the modeler could decide to fix the IIV for some of the parameters to previous estimates based on pooled data from induced blood-stage malaria VIS studies. By default, the models assumed independence of the parameters in their uncertainty and IIV distributions. If, during the model exploration, indications appear of a strong correlation between specific pairs of parameters, both, in their uncertainty and/or IIV distributions, the modeler could attempt to explicitly estimate such parameter correlations. Data below the limit of quantification were included in the dataset and flagged as censored observations.

The following parameters were determined from the population fits of the data:

- Parasite growth rate expressed as the parasite multiplication rate (PMR) standardized to 48 h (PMR_{48}), i.e., ratio of the parasite density at a specific time point to the parasite density 48 h earlier. Based on the population estimates for the GR parameter in the log-linear mixed effect model, PMR_{48} will be reported in log_{10} units:

$$PMR_{48} = \frac{48GR}{\log(10)}$$

- If cycles are observed and if their estimated period is not 48 h, parasite growth rate expressed as the PMR per life cycle (PMR_{LC}). Based on the population estimates for the GR

and P_{len} parameters in the extended log–linear mixed effect model, PMR_{LC} will be reported in $\log_{10}$ units:

$$PMR_{LC} = \frac{P_{len}GR}{\log(10)}$$

- Predicted time to reach parasitaemia thresholds of first positive PCR, ≥5,000 parasites/mL blood for cohorts 1 and 2. All these quantities should be calculated at the population level by solving for t the linear equations: $logX = logParasitaemia_0 + GR \times t$

where $X \in \{250, 5000\}$ and $logParasitaemia_0$ and GR are the population estimates for parasitemia at time of inoculation and parasitemia growth rates respectively.

To determine confidence intervals for the population mean, parameters were sampled from uncertainty.

Blood-stage parasite clearance after antimalarial drug administration

To characterize the blood-stage parasite clearance profile of artemether-lumefantrine in malaria infection in malaria-naïve healthy participants after *Pf*SPZ-DVI challenge; a log–linear model was fitted to the parasitemia data observed from the administration of the treatment onwards. For each participant i:

$$\log_{10} Parasitaemia_{ij} = \beta_{0,i} + \beta_{1,i}t_{ij} + \varepsilon_{ij}$$

where t_{ij} is the time in hours since administration of antimalarial treatment, $\beta_{0,i}$ and $\beta_{1,}$ are the participant's intercept and slope, respectively, and ε_{ij} the error terms. For each participant i, $\beta_{0,}$ is the $\log_{10}$(Parasitaemia at initiation of antimalarial treatment) and $-\beta_{1,i}$ is the clearance rate constant.

The following algorithm was used to remove potential lag and/or tail phases of the parasitemia clearance profile, in order to determine the optimal log-linear decay regression (Marquart et al, 2015). The algorithm considers removing parasitemia data points in an iterative process from both ends of the parasitemia curve, i.e. a combination of removing values from the tail phase and removing values from the lag phase. The optimal log–linear regression model for a participant was deemed an appropriate fit if the overall model p-value was <0.001.

The following parameters were calculated from the estimates of the optimal linear regression model for participant i:

- $\log_{10}$ parasite reduction ratio per 48 h ($\log_{10}$ PRR_{48h}), i.e., ratio of the parasite density at a specific time point to the parasite density 48 h later and expressed in $\log_{10}$.

$$\log_{10}\mathrm{PRR}_{48_i} = -48\beta_{1,i}$$

- Parasite clearance half-life (PC_{50}), i.e., time taken for the parasite density to be reduced by 50% after the first dose administration of artemether-lumefantrine.

$$PC50_i = \log_{10}(2)\left(\frac{48\ hours}{\log_{10}\mathrm{PRR}_{48_i}}\right) = \frac{\log_{10}(2)}{-\beta_{1,i}}$$

- Predicted time to reach parasite clearance of 99% (PC_{99}) was calculated solving for t the linear equation.

$$\log_{10} X = \hat{\beta}_{0,i} + \hat{\beta}_{1,i} \times t$$

where $\hat{\beta}_{0,i}$ is the participant's estimated $\log_{10}$(parasitemia at initiation of artemether-lumefantrine), $\hat{\beta}_{1,i}$ the opposite of clearance rate constant, and X the parasitemia at initiation of artemether-lumefantrine / 100. Thus:

$$PC99_i = \frac{\log_{10}(100)}{-\beta_{1,i}}$$

Marquart, LC, Baker, M, O'Rourke, P, & McCarthy, JS. 2015. Evaluating the pharmacodynamic effect of antimalarial drugs in clinical trials by quantitative PCR. Antimicrob Agents Chemother; 59(7): 4249-59.

Supplemental Table S2: Reasons for screening failure.

Reason	Number
Beck depression score	1
Blood pressure	2
Body mass index and blood pressure	1
Concomitant medication	1
Consent withdrawn	3
Positive drug screen	2
Electrocardiogram abnormal	4
Group postponed	5
Laboratory safety findings abnormal	36
Medical history	1
No longer available	1
Unsuitable veins	4
Verified clinical trial[a]	2

[a]Identified via a commercial database as professional participants or enrolled in multiple trials.

Supplemental Table S3: Demographic and baseline characteristics.

Characteristic	Cohort 1 (N=8)	Cohort 2 (N=8)	All participants (N=16)
Sex			
Males, n (%)	5 (62.5)	5 (62.5)	10 (62.5)
Females, n (%)	3 (37.5)	3 (37.5)	6 (37.5)
Mean age (SD) [range], years	41.6 (9.3) [22, 52]	43.1 (9.2) [32, 54]	42.4 (9.0) [22, 54]
Mean weight (SD) [range], kg	72.4 (12.7) [53.6, 86.8]	79.5 (9.3) [65.6, 95.7]	75.9 (11.4) [53.6, 95.7]
Mean BMI (SD) [range], kg/m^2	24.4 (4.1) [19.0, 29.8]	26.1 (2.4) [21.4, 29.0]	25.3 (3.4) [19.0, 29.8]

All participants self-identified as white race.
BMI, body mass index.

Supplemental Table S4: Participants with treatment-emergent laboratory abnormalities (safety population).

Laboratory parameter, n (%)	Abnormality	Challenge			Rescue			Post-baseline		
		Cohort 1 N=8	Cohort 2 N=8	Total N=16	Cohort 1 N=8	Cohort 2 N=8	Total N=16	Cohort 1 N=8	Cohort 2 N=8	Total N=16
Alanine transaminase, U/L	High	0	0	0	3 (37.5)	7 (87.5)	10 (62.5)	3 (37.5)	7 (87.5)	10 (62.5)
Aspartate transaminase, U/L	High	0	0	0	2 (25.0)	5 (62.5)	7 (43.8)	2 (25.0)	5 (62.5)	7 (43.8)
Protein, g/L	Low	4 (50.0)	2 (25.0)	6 (37.5)	3 (37.5)	1 (12.5)	4 (25.0)	4 (50.0)	2 (25.0)	6 (37.5)
C-reactive protein, mg/L	High	2 (25.0)	1 (12.5)	3 (18.8)	7 (87.5)	8 (100)	15 (93.8)	7 (87.5)	8 (100)	15 (93.8)
Lactate dehydrogenase, U/L	High	0	0	0	5 (62.5)	6 (75.0)	11 (68.8)	5 (62.5)	5 (62.5)	10 (62.5)
Bicarbonate, mmol/L	High	1 (12.5)	1 (12.5)	2 (12.5)	2 (25.0)	7 (87.5)	9 (56.3)	2 (25.0)	7 (87.5)	9 (56.3)
Urate, µmol/L	Low	1 (12.5)	0	1 (6.3)	1 (12.5)	5 (62.5)	6 (37.5)	1 (12.5)	5 (62.5)	6 (37.5)
Potassium, mmol/L	High	0	1 (12.5)	1 (6.3)	3 (37.5)	1 (12.5)	4 (25.0)	3 (37.5)	2 (25.0)	5 (31.3)
Monocytes/leukocytes, %	High	4 (50.0)	2 (25.0)	6 (37.5)	5 (62.5)	6 (75.0)	11 (68.8)	6 (75.0)	6 (75.0)	12 (75.0)
Reticulocytes/erythrocytes, %	High	3 (37.5)	2 (25.0)	5 (31.3)	7 (87.5)	4 (50.0)	11 (68.8)	7 (87.5)	4 (50.0)	11 (68.8)
Leukocytes, 10^9/L	Low	2 (25.0)	0	2 (12.5)	4 (50.0)	5 (62.5)	9 (56.3)	5 (62.5)	5 (62.5)	10 (62.5)
Monocytes, 10^9/L	High	3 (37.5)	6 (75.0)	9 (56.3)	2 (25.0)	4 (50.0)	6 (37.5)	3 (37.5)	6 (75.0)	9 (56.3)
Neutrophils, 10^9/L	Low	1 (12.5)	1 (12.5)	2 (12.5)	5 (62.5)	4 (50.0)	9 (56.3)	5 (62.5)	4 (50.0)	9 (56.3)
Platelets, 10^9/L	Low	2 (25.0)	0	2 (12.5)	4 (50.0)	5 (62.5)	9 (56.3)	4 (50.0)	5 (62.5)	9 (56.3)
Reticulocytes, 10^9/L	High	4 (50.0)	1 (12.5)	5 (31.3)	5 (62.5)	4 (50.0)	9 (56.3)	5 (62.5)	4 (50.0)	9 (56.3)
Eosinophils, 10^9/L	Low	3 (37.5)	2 (25.0)	5 (31.3)	2 (25.0)	3 (37.5)	5 (31.3)	4 (50.0)	4 (50.0)	8 (50.0)
Lymphocytes, 10^9/L	Low	1 (12.5)	2 (25.0)	3 (18.8)	3 (37.5)	2 (25.0)	5 (31.3)	4 (50.0)	3 (37.5)	7 (43.8)
Hematocrit, fraction of 1	High	3 (37.5)	3 (37.5)	6 (37.5)	0	1 (12.5)	1 (6.3)	2 (25.0)	4 (50.0)	6 (37.5)
Basophils/leukocytes, %	High	2 (25.0)	2 (25.0)	4 (25.0)	2 (25.0)	3 (37.5)	5 (31.3)	2 (25.0)	3 (37.5)	5 (31.3)
Hematocrit, fraction of 1	Low	2 (25.0)	2 (25.0)	4 (25.0)	1 (12.5)	2 (25.0)	3 (18.8)	2 (25.0)	2 (25.0)	4 (25.0)
Neutrophils/leukocytes, %	Low	3 (37.5)	1 (12.5)	4 (25.0)	3 (37.5)	1 (12.5)	4 (25.0)	3 (37.5)	1 (12.5)	4 (25.0)
Prothrombin time, seconds	High	3 (37.5)	1 (12.5)	4 (25.0)	2 (25.0)	2 (25.0)	4 (25.0)	4 (50.0)	3 (37.5)	7 (43.8)
Urine erythrocytes, 10^6/L	High	1 (12.5)	2 (25.0)	3 (18.8)	1 (12.5)	0	1 (6.3)	2 (25.0)	2 (25.0)	4 (25.0)
Urine leukocytes, 10^6/L	High	0	2 (25.0)	2 (12.5)	1 (12.5)	2 (25.0)	3 (18.8)	1 (12.5)	3 (37.5)	4 (25.0)

Laboratory abnormalities observed in ≥25% of participants, worse case values following *Pf*SPZ-DIV before antimalarial drug treatment (challenge), following treatment with artemether-lumefantrine (rescue) and overall (post-baseline).

Supplemental Table S5: Participants with treatment-emergent vital sign abnormalities (safety population).

Laboratory parameter, n (%)	Abnormality	Challenge			Rescue			Post-baseline		
		Cohort 1 N=8	Cohort 2 N=8	Total N=16	Cohort 1 N=8	Cohort 2 N=8	Total N=16	Cohort 1 N=8	Cohort 2 N=8	Total N=16
Temperature	High	0	1 (12.5)	1 (6.3)	5 (62.5)	8 (100)	13 (81.3)	5 (62.5)	8 (100)	13 (81.3)
Supine heart rate	High	0	0	0	1 (12.5)	4 (50.0)	5 (31.3)	1 (12.5)	4 (50.0)	5 (31.3)
Supine systolic blood pressure	Low	0	0	0	2 (25.0)	1 (12.5)	3 (18.8)	2 (25.0)	1 (12.5)	3 (18.8)
Supine diastolic blood pressure	Low	0	0	0	1 (12.5)	0	1 (6.3)	1 (12.5)	0	1 (6.3)

Worse case values following *Pf*SPZ-DIV before antimalarial drug treatment (challenge), following treatment with artemether-lumefantrine (rescue) and overall (post-baseline).

Supplemental Table S6: Changes in corrected QT interval from electrocardiograph recordings.

Method	Time post rescue treatment	QTc, ms	Cohort 1 (N=8)	Cohort 2 (N=8)	Overall (N=16)
QTcB	24 hours	>60	0	0	0
		>30 ≤60	0	2 (25.0)	2 (12.5)
		≤30	8 (100)	6 (75.0)	14 (87.5)
		Total	8 (100)	8 (100)	16 (100)
	48 hours	>60	0	0	0
		>30 ≤60	0	1 (12.5)	1 (6.3)
		≤30	8 (100)	7 (87.5)	15 (93.8)
		Total	8 (100)	8 (100)	16 (100)
	Worst case post-baseline	>60	0	0	0
		>30 ≤60	0	2 (25.0)	2 (12.5)
		≤30	8 (100)	6 (75.0)	14 (87.5)
		Total	8 (100)	8 (100)	16 (100)
QTcF	24 hours	>60	0	0	0
		>30 ≤60	0	0	0
		≤30	8 (100)	8 (100)	16 (100)
		Total	8 (100)	8 (100)	16 (100)
	48 hours	>60	0	0	0
		>30 ≤60	0	0	0
		≤30	8 (100)	8 (100)	16 (100)
		Total	8 (100)	8 (100)	16 (100)
	Worst case post-baseline	>60	0	0	0
		>30 ≤60	0	0	0
		≤30	8 (100)	8 (100)	16 (100)
		Total	8 (100)	8 (100)	16 (100)

Values are n (%).
QTcB, QT interval corrected using Bazett's formula; QTcF, QT interval corrected using Fridericia's formula.
